# Modulation of Irisin and Physical Activity on Executive Functions in Obesity and Morbid obesity

**DOI:** 10.1038/srep30820

**Published:** 2016-08-01

**Authors:** A. B. Fagundo, S. Jiménez-Murcia, C. Giner-Bartolomé, Z. Agüera, S. Sauchelli, M. Pardo, A. B. Crujeiras, R. Granero, R. Baños, C. Botella, R. de la Torre, J. M. Fernández-Real, J. C. Fernández-García, G. Frühbeck, A. Rodríguez, N. Mallorquí-Bagué, S. Tárrega, F. J. Tinahones, R. Rodriguez, F. Ortega, J. M. Menchón, F. F. Casanueva, F. Fernández-Aranda

**Affiliations:** 1 Department of Psychiatry, University Hospital of Bellvitge-IDIBELL, Barcelona, Spain; 2 CIBER Fisiopatología Obesidad y Nutrición (CIBERObn), Instituto Salud Carlos III, Spain; 3 Department of Clinical Sciences, School of Medicine, University of Barcelona, Spain; 4 Endocrine Division, Complejo Hospitalario U. de Santiago, Santiago de Compostela University, Spain; 5 Departament de Psicobiologia i Metodologia, Universitat Autònoma de Barcelona, Spain; 6 Department of Pyschological, Personality, Evaluation and Treatment of the University of Valencia, Spain; 7 Department of Basic Psychology, Clinic and Psychobiology of the University Jaume I, Castellón, Spain; 8 Human Pharmacology and Clinical Neurosciences Research Group, Neuroscience Research Program, IMIM-Hospital del Mar Research Institute, Parc de Salut Mar, Barcelona, Spain; 9 Service of Diabetes, Endocrinology and Nutrition, Institut d’Investigació Biomèdica de Girona (IdlBGi) Hospital Dr Josep Trueta, Girona, Spain; 10 Service of Diabetes, Endocrinology and Nutrition, Hospital Clínico Universitario Virgen de Victoria, Málaga, Spain; 11 Department of Endocrinology and Nutrition, University of Navarra, Pamplona, Spain; 12 CIBER Salud Mental (CIBERSAM), Instituto Salud Carlos III, Spain

## Abstract

Whether the executive profile is different between obesity (OB) and morbid obesity (MO) remains unclear. Recent evidence suggests that physical activity (PA) can act as a cognitive enhancer. Irisin is a recently discovered hormone associated with some of the positive effects of PA. The objective of the study was to investigate the executive profile in OB and MO, and to explore the role of PA and irisin. 114 participants were included (21 OB, 44 MO and 49 healthy controls-HC) in the study and assessed with the Wisconsin Card Sorting Test, Stroop Color and Word Test, and Iowa Gambling Task. All participants were female, aged between 18 and 60 years. Results showed a similar dysfunctional profile on decision making in OB and MO compared with HC. Thus, no specific neuropsychological profiles between OB and MO can be clearly observed in our sample. However, a negative correlation was found between irisin and executive functioning. These results demonstrate a specific executive profile in OB and a relevant and negative modulation of irisin on executive functioning. Although irisin might be a promising target for the treatment of obesity, its effects on cognition might be considered when thinking about its therapeutic use.

Obesity has been associated with important biological and environmental risk factors^1^. It has been demonstrated that these patients have a dysfunctional neural pattern, characterized by an alteration in the brain circuitry associated with the reward system^2^,^3^,^4^. From a neuropsychological perspective, deficits in attention, memory and mainly executive functions are major domains defining this pathology^5^. In a previous study, we have demonstrated a dysfunctional executive profile in obesity, mainly in the cognitive flexibility and decision making domains^6^. However, whether this cognitive profile differs between obese (BMI = 30–39.9 kg/m2), and morbid obese subjects (BMI ≥ 40 kg/m2) remains unclear.

The mechanisms underlying the cognitive deficits in obesity are also poorly understood. Some hypotheses, concerning vascular and metabolic states associated with obesity, have been considered as influencing cognitive performance in obesity^7^,^8^,^9^. However, it is well-accepted that cognitive functions are complex and are influenced by a number of social and environmental factors^10^. In this line, recent evidence in both human and animal studies suggests that exercise can act as a cognitive enhancer^11^,^12^,^13^. Of interest, the most important effects of physical activity appear to be on tests measuring executive functions such as response inhibition, working memory and decision making^14^.

Specifically, meta-analyses in humans indicate that physical exercise correlates positively with cognitive performance in healthy subjects and in patients with dementia or mild cognitive impairment^14^,^15^,^16^. Recent neuroimaging studies also suggest that a combination of omega-3 fatty acids, aerobic exercise and cognitive stimulation prevents a decline in the gray matter volume of the frontal, parietal and cingulate cortex in patients with mild cognitive impairment^17^. Particularly interesting are those studies suggesting that physical activity, a balanced diet, cognitive stimulation or the management of conditions such as diabetes and obesity are preventive factors in Alzheimer’s disease^18^. Alongside pharmacological research, these approaches may help slow down the progression of the disease. However, in order to develop successful treatments that complement these strategies, the understanding of the underlying mechanisms is essential.

It has been postulated that irisin, a recently discovered hormone, might be associated with some of the effects of physical exercise such as energy expenditure and thermogenesis^19^, even though its role is far from being clear. Irisin is secreted after the cleaving of the membrane protein fibronectin type III domain containing 5 (FNDC5) and is related to the conversion of white adipose tissue into beige adipose tissue by means of its action in the expression of the uncoupling protein 1 (UCP1). Animal studies suggest that increased plasma levels of irisin are associated with an increase in energy expenditure^19^. In humans, irisin has been linked to body mass index (BMI) despite results being inconsistent. Some authors have reported a positive correlation between circulating irisin levels and BMI^20^,^21^,^22^,^23^, whereas others a negative correlation^24^,^25^. Some variables that can explain these differences are the methods used for measuring circulating levels of irisin and the presence of diabetes. Our results could be crucial since all published findings regarding irisin, and particularly circulating irisin levels, are actually under a cloud of suspicion^26^,^27^. At this point, as we have previously stated in a recent review^28^, there is no doubt as to the need to discern which fraction of FNDC5 is cleaved to produce the soluble portion of irisin. For these reasons, in this study we used the Phoenix pharmaceuticals ELISA kit Ref. EK-067-52, being the only available kit that has been validated^20^. Regardless, based on these findings, it might be postulated that irisin could be a target hormone in conditions characterized by pathological food intake and extreme BMI, such as obesity.

Additionally, previous reports have evidenced that irisin may have a role in the nervous system. This conclusion is based on the expression of FNDC5 in the brain^29^, as well as the presence of irisin in human cerebrospinal fluid and hypothalamic sections^30^. Thus, although it is currently unknown whether irisin can cross the blood-brain barrier and whether irisin may function as a messenger between the skeletal muscle or adipose tissue and brain, previous studies demonstrated that, for example, peripheral administration of irisin is able to reduce blood pressure induced by sympathetic out-flow^31^. Thus, considering the association between irisin levels and physical activity, it is reasonable to consider the role of this hormone on the cognitive effects of exercise in humans, especially taking into account their expression in the brain, as found in several animal studies^29^,^32^.

Thus, the objective of the study was to investigate differences in the cognitive profile of obese subjects versus that of morbid obese subjects, mainly in executive functioning (decision making, response inhibition and cognitive flexibility). We also aimed at exploring the relationship between levels of physical activity and circulating levels of irisin and prefrontal-mediated executive functions in these patients. We hypothesized that both obese and morbid obese subjects would show alterations in executive performance compared to healthy controls. We also hypothesized a role of irisin levels on this dysexecutive profile. For this purpose, we used three neuropsychological tasks (Wisconsin Card Sorting Test; Stroop Color and Word Test; and Iowa Gambling Task) known to be mediated by the prefrontal and orbitofrontal cortex functioning^33^.

## Results

### Sample characteristics

Table 1 includes the descriptive data of the participants of the study. Statistically significant differences were observed across all variables. HC were the group with the highest proportion of university-level education while obese group presented the highest proportion of low academic levels. Mean age was statistically equal for OB and MO (*p *= 214) but patients of these two groups were statistically older than HC (*p* < 001 for both pairwise comparisons). Means for BMI and body-fat were higher for MO, followed by OB and HC (for these two variables linear and quadratic trends achieved significant results indicating that differences between MO vs OB were lower than those obtained for HC vs OB).

### Group comparison for plasma irisin and activity levels

**Figure 1:**
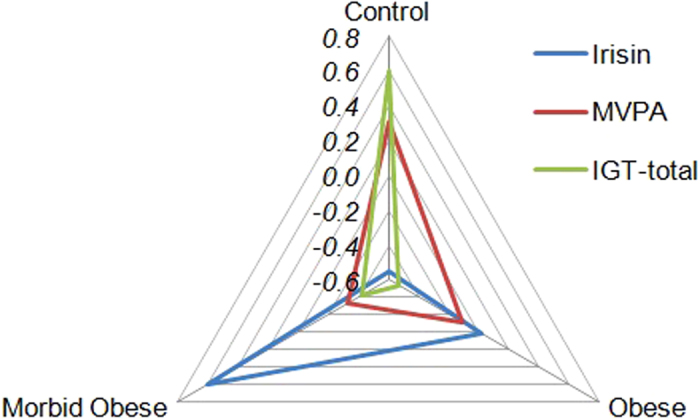
Radar-chart for the distribution of the mean levels of irisin, activity and IGT-total measure in the three groups of weight (Z-scores). *Radar chart displays multivariate results in a two-dimensional chart, representing different axes for each category/group starting from the same point (center of the graphic) and extending outward from the center. Each color-line represents a different variable, and the line size drawn connecting the data to each axis represents the relative magnitude of the variable for the group. So, this graph displays what groups-observations are most similar/different and the connected pattern allows seeing the relative position of each group compared to others. To plot z-scores instead of raw-scores each metric gets equal weight and to simplify the interpretation.

Table 2 contains the comparison between weight groups for circulating levels of irisin and activity levels (MVPA), adjusted by the covariates participants’ age and years of education. Regarding plasma irisin levels, a significant linear trend was obtained (means for irisin tended to increase comparing HC, OB and MO), but a quadratic trend was not statistically significant (mean difference for HC vs OB is similar to mean difference between OB and MO). All pairwise comparisons for plasma irisin levels were statistically significant and obtained moderate to high effect size (|*d*| > 0.50). With respect to activity levels, a linear trend was also achieved (means for MVPA tended to decrease comparing HC, OB and MO) and a quadratic trend was not significant. For MVPA, the only significant pairwise comparison with moderate effect size was that of MO versus HC. Figure 1 shows the radar chart for the means of the z-scores of irisin and activity levels, illustrating that the morbidly obese group was characterized by the highest irisin levels and low MVPA-IGT levels, obese patients by medium MVPA-irisin levels and low IGT measures, while the control group by high IGT scores, medium MVPA and very low irisin levels.

### Group comparison for neuropsychological performance

Table 3 depicts the ANOVA results comparing neuropsychological test performance between weight groups (HC, OB and MO), adjusted by patients’ age and years of education. Only the total IGT scores were different across the three diagnostic conditions: means tended to decrease when comparing HC versus OB and MO (significant linear trend) and the statistical quadratic trend indicates that the difference comparing HC versus OB (means 31.7 vs −0.81) was higher than the difference of OB versus MO (means −0.81 vs 3.40). Pairwise comparisons achieved significant results and a high effect size for HC compared to OB and MO, but no statistical differences were found upon comparing OB and MO. Figure 1 also included the distribution of means for z-IGT scores. No significant differences between groups were observed for the WCST. Lastly, the OB subjects displayed the worse performance in Stroop test (interference score), although no significant differences between the three groups were observed.

### Association between irisin and activity levels with neuropsychological measures

Table 4 contains the partial correlations, adjusted by participants’ age and years of education, between irisin and MVPA with the cognitive outcomes. The r-coefficients were estimated separately (stratified) according to weight group. For HC, activity levels correlated positivity with the WCST-total correct items and negatively with the IGT total score, while irisin correlated negatively with interference and WCST-categories completed and positively with WCST-total errors. For OB, activity levels correlated positively with the IGT-total score and negatively with WCST total correct hits, conceptual answers and categories completed. No association was found between irisin levels and cognitive measures. For MO, the activity levels correlated positively with the interference measures (Stroop interference score) and negatively with the IGT-total score. Irisin levels also correlated negatively with the IGT total score.

## Discussion

This study set out to examine the executive profile of obese and morbidly obese subjects and the role of irisin and activity on prefrontal-dependent cognitive functions in healthy controls and obese subjects. The primary finding of this study was the similar executive profile observed between obese and morbidly obese subjects. According to our results, neither obese nor morbidly obese patients showed significant impairment in the cognitive flexibility capacity or the inhibition response compared to the healthy controls. However, they showed a significant impairment in the decision making capacity. Finally, although not statistically significant a moderate to high effect size of correlations between levels of irisin and physical activity with neuropsychological measures were found. Although physical activity has been previously related to executive functions^12^, this is to the best of our knowledge, the first time that not only physical activity, but also irisin is to some extent associated with executive functioning in humans.

As for decision making performance, both OB and MO subjects went for choices that result in elevated immediate gains despite important future losses, thus showing a similar level of impairment. This profile has been previously described in obese patients^6^, suggesting that reduced decision making abilities are core characteristics of obesity and result in inadequate self-control^6^. Of interest, a recent systematic review examining the relationship between obesity and cognition concluded that decision making is one of the functions most affected in these patients^34^. From a clinical perspective, it might be postulated that there are rational similarities between OB and MO subjects in terms of decision making performance and day to day eating behavior.

These neuropsychological results are also consistent with the hypothesis of food addiction in obesity^4^,^35^. According to this theory, the neural substrates of the decision making’s deficits in obesity are similar to those found in drug addiction. Specifically, previous results have suggested that executive functions, such as decision making, are modulated by dopaminergic functioning (D1 receptors and D2 receptors) in prefrontal cortex and fronto-subcortical circuits^33^. In human and animal models, it has been demonstrated that D2R downregulation mediates signaling in the striatal indirect circuits associated with prefrontal areas^36^. In addition, impairments in those circuits have been linked to less executive control^37^, thus suggesting that DA’s impaired modulation of these areas might be contributing to the impulsive drug intake seen in addiction^38^,^39^. In support, neuroimaging studies in both drug addicted and obese subjects have evidenced low availability of D2R in the striatum to be associated with reduced activity in the ACC and deficient control over food and drug consumption^40^,^41^, which points to the similar brain deregulation in obesity and addiction.

However, we failed to find an association between obesity and inhibition response or cognitive flexibility. According to our results, the executive profile in obesity is more associated with impairments in ‘hot’ executive functions (related to emotional or motivational processes) than ‘cold’ executive functions (more rational or logical processes)^42^. Our results are not in line with those of other studies, showing alterations in cognitive flexibility and inhibition response in obesity^5^. These differences might be explained by the characteristics of the sample. A relevant strength of our study is the exclusion of patients with eating disorders, such as Binge eating disorder (BED) or Bulima nervosa, and diabetes. It has been broadly demonstrated that obese patients with eating disorders or impaired glucose regulation show deficits in inhibition and flexibility^43^. Furthermore, it has been demonstrated that obese individuals with obesity-related somatic comorbidity (i.e., hypertension, diabetes) perform worse in neurocognitive tasks compared to obese individuals without any somatic disorder^44^. Additionally, neuroimaging studies have demonstrated that individuals with BED have a diminished ability to recruit impulse-control-related brain regions compared with obese subjects without BED. The authors concluded that the observed differences in neural correlates of inhibitory processing in BED relative to OB suggest distinct neurobiological contributions to binge eating as a subgroup of obese individuals^45^. All in all, these discrepant results highlight the importance of controlling for these confounder variables in cognitive studies, and conducting a more comprehensive assessment of inhibition response and cognitive flexibility in obesity.

The effects of activity in executive functions are in line with the positive effects of physical activity in other cognitive functions. It has been demonstrated that exercise positively activates cognitive performance^11^,^12^,^13^. In studies with healthy controls, physical activity has been positively correlated to improvements in executive functions such as cognitive flexibility and working memory^14^,^15^. It has also been demonstrated that older adults that engage in physical exercise show a better performance in reasoning capacity, working memory and speed of processing than those maintaining a sedentary lifestyle^46^. Similarly, physically active retired subjects perform better on a series of cognitive tests than retired individuals who are inactive^47^.

However, our results might indicate that the positive effects of physical activity on cognition would not be mediated by the action of irisin. Elevated levels of irisin were associated with disruption in cognitive flexibility performance and inhibition response in healthy controls and with disruption of decision making capacity in OB. Recent studies have been focusing on irisin as a potential treatment for obesity^48^,^49^. However, according to these results a potential cognitive effect of irisin might be considered when thinking about its therapeutic use. Nevertheless, we should keep in mind that this is a correlation study. Thus, future studies should confirm this hypothesis.

Although it is currently unknown whether irisin can cross the blood-brain barrier and whether irisin may function as a messenger between the skeletal muscle or adipose tissue and brain, previous studies have demonstrated, for example, that peripheral administration of irisin is able to reduce blood pressure induced by sympathetic out-flow^31^. From a neuropsychological perspective, the effects of irisin on executive functions might be explained by its action on some neurotransmitters, such as GABA and BDNF^29^. Irisin has recently been detected in the cerebellar Purkinje cells^29^, specifically in GABAergic cells. Although until now it has not been detected in other brain regions, this study points to a novel cerebral pathway that might also be implicating more cortical regions. Dysfunction of the GABAergic system may contribute to cognitive impairment in humans. Specifically, individuals with Alzheimer’s disease have decreased cerebral GABA in the brain and CSF^50^. Furthermore, GABA levels in human CSF decrease with aging^51^, which has been associated with cognitive impairment. According to these studies, the expression of irisin in the GABAergic brain cells might, to some extent, explain its effects on the central nervous system-mediated functions.

From a molecular perspective, the effects of irisin in the prefrontal-related cognitive functions might be mediated by its association with the PPARγ coactivator-1 α (PGC1α). As explained above the effects of irisin are mediated by its action on the expression of UCP1, regulated by PGC1α^19^. Specifically, higher expression of PGC1α is associated with higher levels of irisin. PGC1α is the main transcriptional regulator of mitochondrial function, associated with neurological deficits and cerebral anomalies^52^,^53^. Of interest, it has been demonstrated that PGC1α deficient mice show a significant brain deficiency^54^. In humans, genetic mutations on genes that affect mitochondrial functions have been found to contribute to the pathogenesis of neurodegenerative diseases such as dementia^55^,^56^. In particular, genes that are expressed in response to PGC-1α are under-expressed in Parkinson’s disease and Lewy body disease patients^56^. It has also been suggested that enhancement of PGC-1α by dietary treatment might benefit cognitive function and synaptic plasticity in Alzheimer’s disease by preventing Aβ production in the brain^53^,^55^,^57^. This positive correlation between PGC-1α and cognition was not observed in our study. One possible explanation could be that increased irisin levels in obese subjects result from physiological compensatory mechanisms that may involve decreased sensitivity to irisin or an attempt to increase glucose sensitivity^28^.

This study has several strengths, including the large sample size. Biological approaches, as applied in the present study, are an additional level of analysis on top of neuropsychological assessment, which provides a practical tool for the study of executive processes. Additionally, our study was specifically designed to comprehensively test the executive dysfunction in obese and morbidly obese subjects by using three well validated executive tasks. However, the results of this study should be interpreted in the context of some limitations. First, measures of intelligence quotient (IQ) were not considered, which might have influenced executive performance. Nonetheless, years of education, as a measure of cognitive capacity have been considered in the statistical analysis. Second, only females were included in the study, making the results not applicable to males. Replication of these results with a group including males should be considered. Future studies should also consider including further decision-making, inhibition response and cognitive flexibility tasks in order to shed more light on the mechanisms underlying the executive functions profile in obesity. Moreover, the cross-reactivity of the ELISA immunoassay on detecting irisin FNDC5 precursor at circulating level should be taken into account^28^. Also, although we have used multivariate methods to control the potential biases due to potential confounder variables^58^, the control group is not matched for age and education. Future studies should replicate this study with a control group matched for age and education. Additionally, results reflect moderate to high effect size of correlations between levels of irisin, physical activity and cognition. Future studies should further explore these associations with bigger sample populations to check for p-value statistical significances. Finally, altered irisin levels have been associated with fatty liver, renal, and autoimmune diseases^59^. Future studies including these variables should also be considered.

In summary, our results provide novel information regarding the executive pattern of obesity and the influence of the physical activity and irisin on the cognitive profile. This study is particularly timely given the important public-health impact of obesity and the potential significance of accurately-defined biological variables that are associated. Efforts should focus on determining new biomarkers in order to accelerate the detection of potential drug targets and the implementation of new therapeutic approaches. According to our results, the undesirable effects of irisin on cognition should be considered before using it as a target for the treatment of these extreme eating/weight conditions.

## Material and Methods

### Sample

Seven centers, all involved in the CIBERobn Spanish Research Network, participated: the Eating Disorders Unit (Department of Psychiatry, University Hospital of Bellvitge-IDIBELL, Barcelona), the Department of Endocrinology at the University Hospital of Santiago (Santiago de Compostela); the Department of Diabetes, Endocrinology and Nutrition (Clinic University Hospital Virgen de Victoria, Málaga); the Department of Endocrinology and Nutrition (University of Navarra, Pamplona); the Diabetes, Endocrinology and Nutrition Department, Biomedical Research Institute of Girona (IdIBGi-Doctor Josep Trueta Hospital, Girona); the IMIM (Hospital del Mar Medical Research Institute, Barcelona) and the Department of Basic Psychology, Clinic and Psychobiology (University Jaume I, Castelló). Enrolment into the study took place between January 2010 and September 2013.

One hundred and fourteen participants were included, distributed along the BMI continuum: 21 obese subjects (BMI = 30–39.9 kg/m^2^), 44 morbid obese subjects (BMI ≥ 40 kg/m^2^) and 49 healthy controls (BMI = 18.97–24.61 kg/m^2^). BMI was calculated as the body mass divided by the squared height of the subject. Height was calculated using a stadiometer. All participants were female, aged between 18 and 60 years and spoke Spanish as their first language. Participants were informed about the research procedures and gave informed consent in writing. Procedures were approved by the Ethical Committee of each of the aforementioned institutions (Ref. 048/10; 307/06; 2010/3914/I; 110/2010). The methods were carried out in accordance with the approved guidelines.

The exclusion criteria were: (1) History of chronic medical illness or neurological condition that might affect cognitive function; (2) Head trauma with loss of consciousness for more than 2 min, learning disability or mental retardation; (3) Use of psycho-active medications or drugs (4) Being male; (5) Age under 18 or over 60 (to discard neuropsychological deficits associated with age); (6) Having diabetes type I or II. (7) Obese patients who have comorbid binge eating disorder (DSM-IV criteria^60^). Healthy controls were recruited through several sources including word-of-mouth and advertisements in the local university. Prior to assessment, HC were asked about lifetime or current presence of an eating disorders or obesity. The lifetime history of health or mental illnesses profile was based on the general health questionnaire GHQ-28^61^.

### Neuropsychological assessment

As described in a previous study^6^, all participants underwent a comprehensive neuropsychological and clinical assessment. The neuropsychological tests were selected to cover various aspects of executive functions including decision making, response inhibition, strategic planning and cognitive flexibility and were administered by a trained psychologist in a single session and in a randomized order. All participants were assessed with the following neuropsychological tests: (a) Wisconsin Card Sorting Test (WCST)^62^, (b) Stroop Color and Word Test (SCWT)^63^ and (c) Iowa Gambling Task (IGT)^64^.

#### Wisconsin Card Sorting Test

The WCST is a classical measure of planning capacity, cognitive flexibility, capacity of shifting among stimulus, and control of impulsive responses not aimed at achieving an objective. Subjects have to match a target card with one of four category cards: a single red triangle, two green stars, three yellow crosses, and 4 blue circles. Cards might be matched by color, number, or shape. After each trial a feedback is given to the participant, indicating if they have correctly matched the card. However, along the task the classification rule is unpredictably changing. The test ends when the participant has completed 6 categories or 128 trials. For the purpose of this study the following measures of the test were considered: Total corrects responses, Total errors, Conceptual answers, and Total Categories completed.

#### Stroop Color and Word Test

This paper and pencil test has shown adequate reliability and construct validity for the assessment of inhibition and switching skills. The SCWT measures interference control, flexibility and attention. The task included three pages: (1) a page with color words printed in black ink; (2) a page with “Xs” printed in color; (3) a page with names of colors printed in an incongruent color (i.e. word “blue” printed in red ink). Participants have 45 seconds to read as many words as possible in the first page and name the ink in pages 2 and 3. Three scores are obtained after task completion: number of words (W) (page 1), number of colors named “X” (C) (page 2) and number of color-named words (CW) (page 3). With these scores, the CW estimated (CW′) is calculated by using the formula: CW′ = (W*C)/(W + C). An additional “interference score” is obtained with the formula: Interference = CW-CW′. This is the main variable and higher scores in this variable indicate better capacity of inhibition response.

#### Iowa Gambling Task

This computer task evaluates decision-making, risk and reward and punishment value. The subject has to select 100 cards from four decks (A, B, C and D). After each card selection an output is given: gain or a gain and loss of money. Two decks (A and B) are not advantageous as the final loss is higher than the final gain. Decks C and D, however, are advantageous since the punishments are smaller. The final objective of the task is to make the most of profit and gain as much money as possible. This test is scored by subtracting the amount of cards selected from decks A and B from the amount of cards selected from decks C and D. Higher results point to better performance while negative results point to preference for the not advantageous decks.

### Physical Activity assessment

Physical Activity was evaluated with Actiwatch AW7 (Actiwatch AW7; CamNtech Ltd, Cambridge Neurotechnology, Cambridge, UK), a small (3963269 mm), light-weight (10.5 g) accelerometer that measures activity. The Actiwatch is worn on the non-dominant wrist for 6 days (4 weekdays and 1 weekend), from 00:00 hr on day 1 to 00:00 hr on day 7. Physicacl activity data was calculated in the form of activity counts in a 1-minute epoch length over 24 hours. The counts represent the peak intensity of the movement detected by the Actiwatch AW7. Only the data between 7:00 hr and 23:00 hr is analyzed; a data reduction procedure that has been recommended and conducted in previous studies^65^,^66^. No detected movement for 10 or more consecutive epochs (10 minutes) is considered as missing (seen as implausible counts or as periods during which the participant is sleeping). In addition, a minimum of 4 days of wear is used as criterion to accept the case. This is the lower recommended minimum to accurately estimate daily PA in adults^67^. Upon analysis of the data, there were no cases of 4 or less days of wear. The Actiwatch 7 software (CamNtech Ltd) is used to extract the data. For the purpose of this study the time in Moderate-Vigorous Physical Activity (MVPA) was considered. The average amount of time during the day spent in MVPA was calculated using an algorithm proposed by Heil^67^. Employing the activity monitor Actical (Mini Mitter Co., Inc., Bend, OR), another Actiwatch produced by the same manufacturer, which was placed on the ankle, hip and wrist, Heil^67^ developed algorithms to predict activity energy expenditure (AEE) in children and adults. To obtain the cut point for MVPA, the formula, AEE = 0.02013 + (1.282E-5) × HAC (elaborated for wrist worn accelerometers) was used. This yielded a cut point of 848 counts x min^−1^. This value predicts a PA intensity of 3 MET, which corresponds to a brisk walk. The algorithm to predict AEE in children has been used in a previous study to identify MVPA from the wrist-worn Actiwatch AW4 (CamNtech Ltd, Cambridge Neurotechnology, Cambridge, UK), an earlier version of the Actiwatch AW7^67^. This method has been previously used in obese population^68^.

### Irisin quantification method

Blood samples from overall participants were obtained under basal conditions after a 12-h overnight fast. EDTA-plasma and serum of specimen were separated from whole blood and immediately frozen at −80° C until assay.

The quantitative measurement of irisin in human plasma samples was performed using a commercial enzyme-linked immunosorbent assay (ELISA) kit directed against amino acids 31–143 of the FNDC5 protein (Irisin ELISA Kit EK-067-52; Phoenix Pharmaceuticals, INC, CA) according to the manufacturer’s instructions. Absorbance from each sample was measured in duplicate using a spectrophotometric microplate reader at wavelength of 450 nm (Versamax Microplate Reader; Associates of Cape Cod Incorporated, East Falmouth, MA).

### Statistical analysis

Analyses were carried out with SPSS21 for Windows. First, analysis of variance (ANOVA) valued the association between the diagnosis subtype and the neurocognitive measures. Due the ordinal scale for the weight measure (HC, OB and MOB), ANOVA included polynomial contrasts to explore the presence of trends (linear and quadratic). In addition, considering the importance of age and education on the cognitive performance, these two variables were included as covariates in the ANOVA procedures. The effect size for each pairwise comparison was valued through Cohen’s-*d* coefficient (moderate effect size was considered for |*d*| > 0.50 and high for |*d*| > 0.80). Second, the association between physical activity and irisin with cognitive outcomes was assessed with partial correlations, also adjusted by covariates patients’ age and years of academic studies. Due the strong association between *R*-coefficients and significant test (only large *R*-coefficients achieve significant results in small size samples while small *R*-coefficients achieve significant results in large samples), correlations were interpreted as relevant based on their sample size (moderate effect-size was considered for |*R*| ≥ 0.20 and good effect-size for |*R*| ≥ 0.25^69^,^70^.

Due the multiple statistical tests, potential increases in Type I error was controlled through Bonferroni’s method.

## Additional Information

**How to cite this article**: Fagundo, A. B. *et al*. Modulation of Irisin and Physical Activity on Executive Functions in Obesity and Morbid obesity. *Sci. Rep.* **6**, 30820; doi: 10.1038/srep30820 (2016).

## Figures and Tables

**Table 1:** Sociodemographics, irisin and activity levels.

		HC normal-weight (*n* = 49)	Obesity (*n* = 21)	Morbid-Obesity (*n* = 44)	Statistic	*p*
Academic level; *%*	*Primary*	8.2%	70.6%	61.9%	χ^2^_(df:4)_ = 43.65	**<0.001**
	*Secondary*	38.8%	23.5%	31.0%		
	*University*	53.1%	5.9%	7.1%		
Education (years);	*Range*	9 to 20	7 to 20	0 to 20	F_(df:3;110)_ = 35.33	**<0.001**
	*Mean* (*SD*)	18.47 (2.52)	11.76 (3.82)	13.00 (4.70)		
Age (years);	*Range*	19 to 46	26 to 68	22 to 63	F_(df:3;110)_ = 42.31	**<0.001**
	*Mean* (*SD*)	29.04 (6.22)	49.19 (11.69)	42.25 (10.84)		
BMI (kg/m^2^);	*Range*	18.97 to 24.61	30.79 to 39.11	40.35 to 59.52	F_(df:3;110)_ = 624.4	**<0.001**
	*Mean* (*SD*)	21.61 (1.54)	35.52 (2.40)	46.32 (4.91)		
Body-fat (%);	*Range*	18 to 35	34 to 50	38 to 53	F_(df:3;110)_ = 287.3	**<0.001**
	*Mean* (*SD*)	26.57 (4.50)	41.41 (3.84)	46.55 (3.66)		
Irisin (ng/ml);	*Range*	60.5 to 171.7	92.3 to 176.0	68.7 to 219.8	F_(df:3;110)_ = 20.86	**<0.001**
	*Mean* (*SD*)	104.00 (24.32)	122.38 (23.63)	141.40 (32.96)		
Activity: MVPA;	*Range*	14.0 to 154.2	9.0 to 151.5	3.7 to 126.8	F_(df:3;110)_ = 5.04	**0.008**
	*Mean* (*SD*)	71.37 (34.09)	54.47 (43.82)	47.40 (34.15)		

BMI: Body mass index. SD: standard deviation.

**Table 2:** Polynomial trends and pairwise comparisons for Irisin and Physical activity (ANOVA adjusted by covariates participants’ age and years of education).

	Adjusted means					Trends				Pairwise-comparisons: mean differences								
	HC	OB	MO	Factor Group		Linear		Quadratic		OB vs HC			MO vs HC			MO vs OB		
	*n* = 49	*n* = 21	*n* = 44	*F*(_*2;111*)_	*p*	*F*(_*1;111*)_	*p*	*F*(_*1;111*)_	*p*	MD	*p*	*|d|*	MD	*p*	*|d|*	MD	*p*	*|d|*
Irisin (ng/ml)	104.0	122.4	141.4	20.86	0.002	41.72	0.002	0.002	0.999	18.38	0.026	0.77*	37.40	0.002	1.29*	19.02	0.022	0.66*
Activity: MVPA	71.37	54.47	47.40	5.04	0.016	9.74	0.004	0.192	0.999	−15.90	0.198	0.41	−23.97	0.004	0.70*	−8.07	0.830	0.20

Abbreviations: HC, Healthy controls normal-weight; OB, Obesity; MO, Morbid-Obesity; |*d*|: Cohen’s-d. *Moderate to high effect size (|*d*| > 0.50). |*d*|: Cohen’s-*d*. *Moderate to high effect size (|*d*| > 0.50). Results include Bonferronis-correction for multiple comparisons.

**Table 3:** Polynomial trends and pairwise comparisons for neuropsychological variables: ANOVA adjusted by the covariates participants’ age and years of education.

		Adjusted means			Factor		Trends				Pairwise-comparisons: mean differences								
		HC	OB	MO	Group		Linear		Quadratic		OB vs HC			MO vs HC			MO vs OB		
		*n* = 49	*n* = 21	*n* = 44	*F*(_*2;109*)_	*p*	*CE*	*p*	*CE*	*p*	MD	*p*	*|d|*	MD	*p*	*|d|*	MD	*p*	*|d|*
STROOP	Interference	2.52	−1.61	1.27	2.05	0.348	−0.89	0.442	2.86	0.132	−4.13	0.173	0.45	−1.25	0.583	0.19	2.88	0.436	0.46
WCST	Total corrects	64.02	67.49	69.22	1.25	0.497	3.68	0.119	−0.71	0.850	3.48	0.571	0.30	5.20	0.316	0.43	1.73	0.815	0.11
	Total errors	30.13	36.38	34.13	0.47	0.692	2.83	0.454	−3.47	0.676	6.25	0.571	0.29	4.01	0.583	0.18	−2.25	0.815	0.08
	Conceptual answers	54.76	56.65	60.17	0.88	0.555	3.82	0.224	0.66	0.857	1.90	0.759	0.11	5.41	0.398	0.31	3.51	0.815	0.16
	Categories completed	4.64	4.43	4.64	0.13	0.881	0.00	0.997	0.18	0.774	−0.22	0.759	0.12	0.00	0.997	0.01	0.22	0.815	0.09
IGT	Total	31.73	−0.81	3.40	11.24	**0.001**	−20.04	**<0.001**	15.00	**0.041**	−**32.5**	**0.001**	1.28*	−**28.3**	**0.001**	1.20*	4.21	0.815	0.19

Abbreviations: HC, healthy controls normal-weight; OB, Obesity; MO, Morbid-Obesity. SCWT, Stroop Color and Word Test; WCST, Wisconsin Card Sorting Test; IGT, Iowa Gambling Task; Conc.answers, conceptual answers; Cat.complet., categories completed. |*d*|: Cohen’s-d. *Moderate to high effect size (|*d*| > 0.50). Results include Bonferroni’s-correction for multiple comparisons.

**Table 4:** Partial correlations (adjusted by the covariates age and years of education) between cognitive variables, physical activity and irisin.

		HC (*n* = 49)				OB (*n* = 21)				MO (*n* = 44)			
		Activity		Irisin		Activity		Irisin		Activity		Irisin	
		*r*	*p*	*r*	*p*	*r*	*p*	*r*	*p*	*r*	*p*	*r*	*p*
STROOP	Interference	−0.081	0.594	−0.228*	0.128	−0.070	0.781	0.169	0.501	0.311*	0.078	−0.038	0.835
WCST	Total corrects	0.223*	0.136	0.033	0.828	−0.332*	0.179	0.170	0.500	0.154	0.392	0.088	0.624
	Total errors	−0.056	0.711	0.233*	0.119	0.136	0.591	0.159	0.530	−0.057	0.754	−0.148	0.410
	Conceptual answers	0.193	0.200	−0.072	0.632	−0.315*	0.203	−0.091	0.719	0.150	0.405	0.143	0.426
	Categories completed	0.157	0.299	−0.204*	0.173	−0.335*	0.175	−0.083	0.743	0.157	0.384	0.012	0.948
IGT	Total	−0.283*	0.056	0.151	0.317	0.491*	0.038	−0.194	0.440	−0.293*	0.098	−0.335*	0.057

HC, healthy control normal-weight; CO-over., control over-weight; OB, Obesity; MO, Morbid-Obesity. Activity: MVPA score. SCWT, Stroop Color and Word Test; WCST, Wisconsin Card Sorting Test; IGT, Iowa Gambling Task.

^*^Bold: moderate to high effect size for correlation (|*R*| > 0.20). Correlation matrix includes Bonferroni’s correction for multiple comparisons.
